# Antibacterial Activity of Copper Nanoparticles (CuNPs) against a Resistant Calcium Hydroxide Multispecies Endodontic Biofilm

**DOI:** 10.3390/nano11092254

**Published:** 2021-08-31

**Authors:** Beatriz Rojas, Nicole Soto, Marcela Villalba, Helia Bello-Toledo, Manuel Meléndrez-Castro, Gabriela Sánchez-Sanhueza

**Affiliations:** 1 Department of Restorative Dentistry, Faculty of Dentistry, Universidad de Concepción, Concepción 4030000, Chile; beatriz.a.rojas.s@gmail.com (B.R.); nicolestm@gmail.com (N.S.); marcela.villalbam@gmail.com (M.V.); 2 Research Laboratory Antibacterial Agents (LIAA), Department of Microbiology, Faculty of Biological Sciences, Universidad de Concepción, Concepción 4030000, Chile; hbello@udec.cl; 3 Millennium Nucleus for Collaborative Research on Bacterial Resistance (MICROB-R), Santiago 8320000, Chile; 4 Advanced Nanocomposites Research Group (GINA), Hybrid Materials Laboratory (HML), Department of Materials Engineering (DIMAT), Faculty of Engineering, Universidad de Concepción, Concepción 4030000, Chile; mmelendrez@udec.cl

**Keywords:** copper nanoparticles, intracanal medication, antibacterial activity, endodontic biofilm

## Abstract

Endodontic treatment reduces the amount of bacteria by using antimicrobial agents to favor healing. However, disinfecting all of the canal system is difficult due to its anatomical complexity and may result in endodontic failure. Copper nanoparticles have antimicrobial activity against diverse microorganisms, especially to resistant strains, and offer a potential alternative for disinfection during endodontic therapy. This study evaluated the antibacterial action of copper nanoparticles (CuNPs) on an ex vivo multispecies biofilm using plaque count compared to the antibacterial activity of calcium hydroxide Ca(OH)_2_. There were significant differences between the Ca(OH)_2_ and CuNPs groups as an intracanal dressing compared with the CuNPs groups as an irrigation solution (*p* < 0.0001). An increase in the count of the group exposed to 7 days of Ca(OH)_2_ was observed compared to the group exposed to Ca(OH)_2_ for 1 day. These findings differ from what was observed with CuNPs in the same period of time. Antibacterial activity of CuNPs was observed on a multispecies biofilm, detecting an immediate action and over-time effect, gradually reaching their highest efficacy on day 7 after application. The latter raises the possibility of the emergence of Ca(OH)_2_-resistant strains and supports the use of CuNPs as alternative intracanal medication.

## 1. Introduction

One of the principal causes of endodontic failure is the remaining microorganisms in the root canal system [1]. It is well-documented that microorganisms possess and develop resistance to endodontic disinfectant agents, which further complicates the treatment of the root canal system [2]. The problem increases because during infection, microorganisms form biofilms, making them 1000 times more difficult to eliminate [3,4].

Secondary endodontic infections show a high prevalence of *Enterococcus faecalis*, as they are able to persist after root canal treatment. Due to its bactericidal activity, calcium hydroxide (Ca(OH)_2_) is the antimicrobial agent most used for intracanal medication. A central aspect related to the antimicrobial activity of Ca(OH)_2_ is its high pH, close to 12.5. However, some studies have demonstrated that *E. faecalis* can survive even in this basic environment [5,6]. *E. faecalis* is described as the predominant bacterium in root canals associated with persistent periradicular lesions [7,8]. However, recent studies reject this hypothesis, where *Streptococcus mutans* was found in persistent infections, emphasizing the presence of diverse microbiota [9,10].

Copper, a trace element crucial for life, is involved in a broad range of processes and has been used by humans for more than 10,000 years. Recently, copper has become a focus of scientific interest due to its antimicrobial properties and its reported low toxicity in humans. Combined pharmacological complexes based on copper are more effective as antibacterial, antifungal, and antiviral agents [11,12]. The mechanism by which copper acts produces a bacteriostatic or bactericidal effect, which was directly related to its concentration [13]. The maximum reported effect has been for copper metal (99.9%) and these results have been observed in alloys containing at least 70% copper [11,12]. However, owing to the anatomy of the root canal system, which has microecological niches such as dentinal tubules, where antimicrobial agents cannot reach, nanotechnology appears as an alternative to increase the success rate of treatments and endodontic retreatments [14].

Nanoparticles originate from a metal of a macrometric size. They have a diameter smaller than 100 nm with different and improved properties with respect to the original metal [15]. Copper nanoparticles (CuNPs) have various possible uses in medicine, optics, and the electronic field. CuNPs are used in the manufacture of conductive elements, lubrication, nanofluids, and as a stronger antimicrobial agent. CuNPs are effective against Gram-positive and Gram-negative bacteria and fungi [16]. Their antimicrobial action depends on their size. To achieve maximum antibacterial activity, they must be synthesized to a size that allows greater contact of the nanostructure with the bacterial surface, showing a more effective antimicrobial action than at their normal size, covering a broad bacterial spectrum, including multidrug-resistant microorganisms [15].

CuNPs at a concentration of 300 ppm through the reduction of copper salt, with average diameters of 50 nm, were tested against different microorganisms showing high antimicrobial action against Gram-positive bacteria such as methicillin-resistant *Staphylococcus aureus* (MRSA) [17].

CuNPs can penetrate the bacterial cell wall, resulting in cellular damage. In the cell, nanoparticles indirectly alter DNA or protein synthesis, inactivate their enzymes, and promote the generation of hydrogen peroxide [18]. Finally, nanoparticles interact with –SH groups (atoms of hydrogen), which leads to the denaturation of proteins. All these elements make the possibility of selecting resistant strains extremely low [18].

Finding a new antibacterial agent as an alternative for use in endodontic treatment is essential to reduce the resistance of microorganisms. Unfortunately, it is well-known that the systematic application of high doses of an antimicrobial agent leads to the selection of strains that produce higher levels of persistent bacteria. This is precisely what happens in the treatment of chronic infections, which is becoming a serious threat since, in the near future, we may find ourselves deprived of effective antimicrobial agents [19,20].

## 2. Materials and Methods

This project was conducted in accordance with the principles of the Code of Ethics of the Belmont Report, supported by an informed consent and approved by the Ethics Committee of the School of Dentistry for the use of isolated clinical strains from persistent chronic apical periodontitis for an ex vivo biofilm model. (C.I.Y.B. No. 04/15).

### 2.1. Selection of the Sample

Eighty roots from extracted teeth with type I canals according to the Weine classification were selected for the study. Samples had slight moderate curves (less than 20°) according to Schneider, with a minimum of nine millimeters in length. They were clinically permeable, with complete apical closure and stored in physiological serum [14]. To measure the root curvatures, dental X-rays were taken and angular measurements were performed using VistaScan software (Dürr Dental^®^, Stutgart, Germany).

### 2.2. Development of an Ex Vivo Model of Aerobic Artificial Biofilm on Root Canal Surface

Eighty root canals were prepared using the reciprocal instrumentation technique with a 25/08 Wave OnePrimary file (Dentsply- Maillefer^®^, Ballaigues, Switzerland) and constant irrigation with sodium hypochlorite 5.25%, following the manufacturer instructions. After instrumentation, canals were irrigated with a solution of 17% EDTA for 3 min and finally, 5.25% NaOCl to completely remove dentinal smear layer. The roots were dried with sterile swab and covered on the outer surface with 2 layers of nail polish (taking care not to block the entrance to the canal) to avoid external contamination of the roots. Subsequently, samples were taken individually into test tubes with a phosphate-buffered saline, then autoclaved for 30 min at 121 °C. The efficacy of sterilization was confirmed using the protocol proposed by Javidi [14].

Bacterial strains *Streptococcus mutans* American Type Culture Collection (ATCC^®^, Manassas, VA, USA) 25175^TM^ and the isolated root canal strain, *E. faecalis* UDEC 6.1 confirmed by polymerase chain reaction (PCR), were cultured aerobically at 37 °C for 24 h on brain heart infusion agar (BHA, Merck Millipore, Darmstadt, Germany). A colony of each strain was inoculated separately in 5 mL of BHI broth to ensure a pure culture. After 18 to 20 h of incubation at 37 °C, the suspension of each strain was adjusted using an Oxoid turbidimeter (Fisher Scientific Company, Ottawa, ON, Canada) until a turbidity equivalent to McFarland 0.5 was achieved (1.5–2 × 10^8^ CFU/mL). A 500 μL aliquot of each adjusted suspension was deposited in each tube containing the dental specimens. The tubes with the samples were incubated at 37 °C for 48 h to develop a young multispecies biofilm. After incubation, samples were washed gently 2 times with 1 mL PBS to remove detached bacteria. Roots were divided into 9 groups: group 1 was the negative control group (no treatment, irrigation with 5 mL of NaCl 0.9% for 1 min), groups 2 to 6 were treated with CuNPs for 1, 10, 30, 60 min, 1 day, and 7 days, respectively; groups 7 and 8 were the positive control treated with calcium hydroxide (UltraCal^®^ XS) for 1 day and 7 days, respectively.

### 2.3. Dressing and Incubation of the Roots

Medication was applied and samples were taken from each tube according to the respective medication period. Because nanoparticles do not dissolve but disperse in liquid, propylene glycol was used at the concentration of ¼ of the highest minimum inhibitory concentration (MIC) for both strains as a dispersing medium. A dispersion with a concentration equal to the MIC was prepared for *Enterococcus faecalis* UDEC 6.1, corresponding to 256 μg/mL, greater than the MIC for *Streptococcus mutans* ATCC 25175, which was determined as 125 μg/mL [21].

### 2.4. Synthesis and Characterization of Nanoparticles

The synthesis method of CuNPs was a controlled atmosphere arc discharge (CA-ARC) as reported by Medina et al. [22]. High-purity (99.99%) Cu wires 2 mm in diameter (Sulzer, Winterthur, Switzerland) were used as the raw material, and an argon carrier and stabilizer gas with a flow rate of 500 sccm. The operating voltage and current were 25 V and 30 A, respectively. The test was carried out at 1 atm. The reaction chamber and the accumulation chamber were enriched with oxygen prior to discharge. The reaction of the precursors by arc discharge was carried out in pulses to avoid overheating of the reactor. The arc tilt angle was 408 with an inter-electrode distance of 0.5 cm, and the precursor wire velocity was 0.5 cm/s.

A JEM-ARM200F analytical microscope (JEOL Ltd., Tokyo, Japan) with a resolution of 0.08 nm and aberration correction was used to perform high-resolution transmission electron microscopy (HRTEM). A JEM 1200 EX II high-resolution scanning/transmission electron microscope (JEOL Ltd., Tokyo, Japan) operating at 200 kV (0.19 nm point resolution) was also used to perform selected area electron diffraction. Particle size measurements were performed through DigitalMicrograph™, Gatan Inc. (Pleasanton, CA, USA) image acquisition and processing software. The size frequency histogram was performed using Origin 8.0, from OriginLab Corporation (Northampton, MA, USA).

### 2.5. Plaque Count

Following the respective incubation times in the laminar flow chamber (Thermo Scientific^®^, Marietta, OH, USA), samples were taken and evaluated by counting the colony-forming units (CFUs) in triplicate, according to Javidi’s protocol [10]. To verify there was no contamination, colonies were taken randomly on day 7 and observed under a microscope with a 100× magnification and immersion objective.

### 2.6. Statistical Analysis 

To compare the activity of antibacterial agents, non-parametric ANOVA (Kruskal–Wallis test) and the Bonferroni multiple comparison test were performed. The level of significance was 5%. All statistical analyses were carried out using the InfoStat^®^ program.

## 3. Results

**Figure 1:**
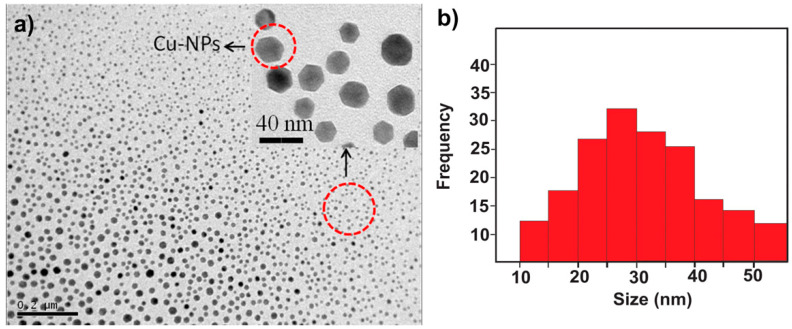
(**a**) Micrograph of copper nanoparticles obtained by arc discharge in a controlled atmosphere; (**b**) particle size distribution histogram of the nanoparticles obtained.

CuNPs were obtained by a new technique known as arc discharge in a controlled atmosphere (DARC-AC). In this technique, no stabilizing agent is used, so the surface of these nanoparticles is not passivated. Passivation of the metal surface causes the electrical, optical, and even antimicrobial properties to worsen because electronic or ionic exchange with the medium is not possible due to stabilization. The obtained particles were preferably spherical (Figure 1a) and had a size that ranged between 20 and 60 nm, as shown in Figure 1b. Most of the structures had well-defined decahedral (mostly spherical) shapes. The low-resolution image shows the low dispersion in size of the nanoparticles.

**Figure 2:**
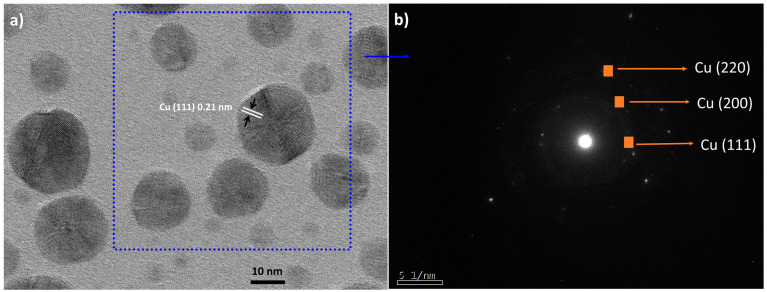
(**a**) High-resolution micrograph of copper nanoparticles obtained by DARC-AC and (**b**) selected area electron diffraction (SAED) of CuNPs.

Figure 2 shows a high-resolution electron micrograph where the slightly more defined morphology of the nanoparticles can be observed. The measurement of the crystalline planes in Figure 2a allowed us to determine an interplanar distance of 0.21 nm that corresponds to the crystalline plane (111) of metallic Cu (FCC). This structure is confirmed by Figure 2b by analysis of selected area electron diffraction (SAED) of CuNPs. The following crystalline planes were determined: (220), (200), and (111), which correspond to metallic copper, corroborating what was found in the HRTEM micrographs. 

**Figure 3:**
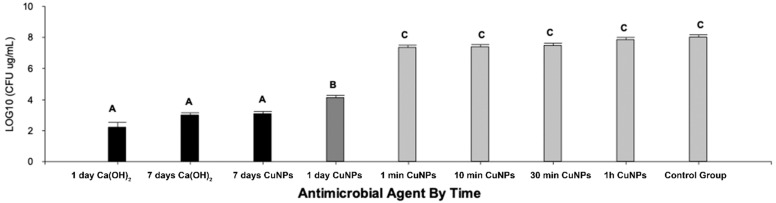
CFU count in log_10_ per group and statistically significant differences between groups A, B, and C. (*p* < 0.0001; ANOVA).

Colonies of *S. mutans* and *E. faecalis*, coming from the biofilm formed, were grown onto plates. After the plate count, for a concentration corresponding to 256 μg/mL of CuNPs, all the groups treated for 1 day or more showed antimicrobial activity since a decrease of more than three logarithms was observed in the count in comparison with the control group. There were significant differences in mean per treatment (*p* < 0.0001) in the Kruskal–Wallis test. Additionally, in the Bonferroni multiple comparisons test, significant differences were obtained in mean per treatment (*p* < 0.0001; ANOVA). The groups with different letters (A, B, and C in Figure 3) showed statistically significant differences. Groups with a common letter were not significantly different (*p* > 0.05).

## 4. Discussion

No phases corresponding to copper oxide were observed, so the particles, despite not having a stabilizing agent, were not oxidized. This is the main advantage of the DARC-AC synthesis, where the argon atmosphere prevents the production of oxide while obtaining metallic nanoparticles [21,23].

Bacterial resistance is a constant concern in the failure of root treatments, hence the importance of studying new antimicrobial agents that can be used as coadjuvants and/or alternatives in endodontic therapy [24]. Copper has multiple properties, among which antimicrobial contact and anti-contact action avoid the appearance of resistant microorganisms and acts as a better antimicrobial dressing than Ca(OH)_2_ in the root canal ex vivo model [25]. CuNPs improve the properties of copper as a pure metal, which is a great advantage as they could access tiny dentinal tubules with an average size of 5 μm, in which the endodontic biofilm is housed [15]. This report showed antimicrobial activity in all tested groups of one day or more, since a decrease of more than three logarithms was observed in four of all pre-established times, which is why, from the microbiological point of view, these groups behaved as bactericidal agents. Statistically significant differences were obtained between samples exposed for one day or more compared to samples exposed at 1 h or less. This reinforces the idea that CuNPs could be an alternative treatment as a medication and not as an adjunct in the irrigation of canals, at least at the concentration used in this study. This was previously described for other antimicrobial nanostructures whose behavior was better as a medication than as an irrigant [16]. Although these results contrast those reported by Javidi et al., they are not comparable since the actual results are expressed in the logarithm of the counts, the unit of measurement accepted for this type of test [14].

Our results suggest there are no significant differences between the antibacterial action of CuNPs at 7 days and Ca(OH)_2_ on 1 day and at 7 days of exposure, similar to another study [14]. However, there are statistically significant differences in these groups with the samples exposed to 1 day of CuNPs. We assume that the antibacterial action was lower in the groups that were exposed for only minutes because the MIC used for CuNPs in this ex vivo study was lower compared to the average MIC for the same species in other in vitro studies [26,27,28,29]. When using in vitro MICs in ex vivo or in vivo tests, there are other factors that affect the efficiency of the antimicrobial agent (biofilm, interaction with other tissues, pH, oxygen, etc.). Therefore, we suggest performing assays with two and four times the MIC for CuNPs in addition to increasing the number of bacterial species to include anaerobes in a mature biofilm, since this will allow obtaining more conclusive results, closer to the in vivo environment of the root canal system [5]. This would give CuNPs greater antimicrobial efficacy over time [27].

In this study, we failed to establish statistically significant differences between CuNPs and Ca(OH)_2_ at 7 days of treatment, resulting in similar CFU counts. Although the count was a larger log on the 1 day exposure to CuNPs compared to the 7 day exposure, from the viewpoint of resistance selection, it is an excellent result compared to Ca(OH)_2_. CuNPs have anticancer activity due to their potential for degradation of DNA, which is in direct relation to their low potential for selection of resistant strains [19]. CuNPs degrade DNA with oxygen mediation, even with any external agent such as hydrogen peroxide or ascorbate. This makes CuNPs ideal candidates for targeted therapy. The use of CuNPs as a treatment agent could be particularly beneficial because the human body has an efficient system for metabolizing copper, since it is a micronutrient, so the residual copper produced can be easily processed [30,31]. CuNPs antimicrobial agents are biocompatible at low antibacterial effective doses and show a cytotoxic effect after more than 6 h of exposure [21].

Additionally, the cytotoxic effect of CuNPs and potential DNA degradation can be used in the formulation of anticancer drugs by chemically modifying copper [15]. The toxicity of CuNPs, which is based especially on their cytotoxic potential, should be considered [32]. For toxicity testing of metal nanoparticles, the dose released (i.e., mass of nanoparticles per volume of suspension) should be considered rather than only the dose administered (initial mass concentration of nanoparticles) [33]. In vitro dose-response outcomes depend on complex toxicodynamic events, including the activation of cellular response pathways involved in nanoparticle uptake, the nanoparticle/cell association ratio, and multiple physicochemical parameters that influence nanoparticle sedimentation and internalization. It is suggested to perform in vivo studies to evaluate how CuNPs and released ions may be involved in the antibacterial mechanism into dentinal tubules at the level of apical constriction, and its relation to the cytotoxicity of CuNPs on the apical tissues [34]. It is important to develop a more realistic experimental design based on a mature multispecies bacterial model, which allows a better understanding of the performance of antimicrobial materials against bacteria [35,36].

## 5. Conclusions

In the present study, it was possible to observe the antibacterial activity of CuNPs on a biofilm of *E. faecalis* and *S. mutans*. It was possible to detect an immediate action and an over-time effect, gradually reaching their highest efficacy on day 7 after application. The latter shows the potential use of CuNPs as intracanal medication. It is important to emphasize the action of calcium hydroxide from a commercial preparation, which was higher on day 1 compared to day 7; this suggests shorter medication periods with superior effectiveness in root canal treatments, reducing selection pressure within the root canal system.

## Data Availability

Not applicable.
